# Genomic characterization of vancomycin-resistant *Enterococcus faecium* and van-carrying mobile genetic elements in a tertiary hospital in northeastern China

**DOI:** 10.3389/fmicb.2026.1804495

**Published:** 2026-04-10

**Authors:** Han Yao, Jiaxin Wang, Rongrong Dong, Wenjing Yi, Meng Zhang, Ning Zhu, Shanshan Jia, Ruihong Wu, Xiaohan Guo, Tingting Dong, Zhihan Peng, Lili Jiang, Wei Li, Chun Yang, Mengyao Yuan, Qingtian Guan, Jiancheng Xu

**Affiliations:** 1 Department of Laboratory Medicine, Infectious Diseases and Pathogen Biology Center, The First Hospital of Jilin University, Changchun, China; 2 Bioinformatics Laboratory, Infectious Diseases and Pathogen Biology Center, The First Hospital of Jilin University, Changchun, China; 3 Department of Respiratory Medicine, Center for Pathogen Biology and Infectious Diseases, The First Hospital of Jilin University, Changchun, China; 4 Department of Core Facility, The First Hospital of Jilin University, Changchun, China; 5 State Key Laboratory for Diagnosis and Treatment of Severe Zoonotic Infectious Diseases, Key Laboratory for Zoonosis Research of the Ministry of Education, Department of Respiratory Medicine, Infectious Diseases and Pathogen Biology Center, The First Hospital of Jilin University, Changchun, China; 6 Health Examination Center, The First Hospital of Jilin University, Changchun, China

**Keywords:** clonal vancomycin resistance, Enterococcus faecium, genome sequencing, genomic epidemiology, horizontal gene transfer

## Abstract

**Background:**

Vancomycin resistant *Enterococcus faecium* (VREfm) poses a significant healthcare challenge due to its multidrug resistance and genomic plasticity. Vancomycin resistance is commonly mediated by van gene clusters located on transposons, which are often associated with plasmids.

**Methods:**

19 VREfm isolates were collected from different departments of the First Hospital of Jilin University between 2019 and 2024. Whole-genome sequencing (WGS) was performed on the strains for comprehensive genomic analysis. Multilocus sequence typing (MLST) was used to determine the sequence types of the strains. Plasmids were grouped based on Mash distance, and plasmid content was analyzed using the MOB-suite tool. Genome-wide comparisons and average nucleotide identity (ANI) analysis were conducted using FastANI. The TnCentral database was used to analyze resistance-associated transposons. The objective was to characterize the genomic diversity of VREfm isolates and the genetic contexts of van-carrying plasmids and transposons.

**Results:**

Among the 19 VREfm isolates, *vanA* was detected in 16 isolates, while *vanM* was identified in 3 isolates. MLST analysis revealed five sequence types (ST17, ST68, ST78, ST80, and ST547) with distinct temporal distributions. ST17 and ST68 were more frequently observed among isolates collected between 2019 and 2022, whereas ST78 was more common among isolates collected in 2023–2024 and was associated with multiple plasmid types. These observations suggest differences in lineage composition and plasmid backgrounds across the sampling period. Plasmidome analysis identified 19 plasmid groups, with resistance genes mainly concentrated in four major groups, some of which were shared across different sequence types. Notably, several resistance plasmids lacked functional replicons, suggesting plasmid fragmentation events. Transposon analysis revealed substantial structural diversity among *Tn1546* variants, including insertions, deletions, and rearrangements, highlighting the complexity of *vanA*- and *vanM*-associated mobile genetic elements across different plasmid and clonal backgrounds.

**Conclusion:**

This study provides genomic insights into the diversity and relatedness of VREfm isolates in a tertiary hospital over a 5-year period. The findings describe the diversity of sequence types, plasmid backbones, and van-associated mobile genetic elements within this hospital collection.

## 1 Introduction

*Enterococcus* is a genus of Gram-positive, facultative anaerobic cocci and one of the most common pathogens responsible for nosocomial infections. Among the 87 identified *Enterococcus species* (Parte et al., 2020), *Enterococcus faecalis (E. faecalis)* and *Enterococcus faecium (E. faecium)* are major causes of infection in healthcare settings (Sghir et al., 2000; Weiner et al., 2016). According to the China Antimicrobial Resistance Surveillance System (CARSS) 2023 National Antimicrobial Resistance Surveillance Report, *E. faecium* and *E. faecalis* each account for 10.8% of the top five Gram-positive bacteria by isolation rate. In the USA, *E. faecalis* and *E. faecium* account for 7.4 and 3.7% of all hospital-acquired infections (HAIs) (Weiner et al., 2016). As an opportunistic pathogen, *Enterococcus* can cause severe diseases such as urinary tract infections, infective endocarditis, and bloodstream infections (Werner et al., 2020). They can spread directly between patients or from contaminated surfaces like bed rails, over-bed tables, and door handles (Lee A. S. et al., 2018; McDermott et al., 2018).

The growing antibiotic resistance observed in clinical isolates is a significant challenge in treating enterococcal infections. Penicillin and ampicillin can inhibit the growth of *E. faecium*, but they are unable to eradicate its cells, leading to reduced efficacy ultimately (Baddour et al., 2015). This has increased the reliance on vancomycin, making it the preferred antibiotic for treating *E. faecium* infections. However, the emergence of multidrug-resistant strains, particularly vancomycin-resistant *enterococci* (VRE), which were first identified in the 1980s in Europe (Uttley et al., 1988), has become a critical public health concern due to the widespread use of antimicrobials (Uttley et al., 1988; MacDougall et al., 2020).

In recent years, the vancomycin resistance of *E. faecium* (VREfm) has shown a significant upward trend in China. According to the China Antimicrobial Resistance Surveillance System (CARSS, 2005) annual report, the average vancomycin resistance rate of VREfm increased from 1.0% in 2020 to 1.2% in 2021, 1.7% in 2022, and 2.9% in 2023. Notably, the vancomycin resistance rates of VREfm in the three northeastern provinces (Liaoning, Jilin, and Heilongjiang) have continuously increased over the past four years. In Jilin Province, the resistance rate rose from 0.4% in 2020 to 2.0% in 2021, 2.4% in 2022, and increased to 3.7% in 2023. Similarly, Liaoning Province experienced an increase from 1.3% in 2020 to 2.2% in 2021 and 2022, followed by a sharp rise to 5.6% in 2023. In Heilongjiang Province, the resistance rate fluctuated from 0.9% in 2020 to 0.4% in 2021, then increased to 1.4% in 2022 and rose to 3.1% in 2023. This persistent upward trend suggests that the proportion of VREfm in the northeastern provinces is steadily increasing, which may be closely associated with changes in local antibiotic usage patterns and healthcare environments.

*Van* genes primarily mediate vancomycin resistance in *Enterococcus*. To date, nine *van* gene clusters have been identified: *vanA, vanB, vanC, vanD, vanE, vanG, vanL, vanM, and vanN (Ahmed and Baptiste, 2018). *VanA* and *vanB*, which are associated with the transposon sequences *Tn1546* and *Tn1549*, respectively, represent the most prevalent and clinically significant vancomycin resistance gene cluster (Guzman Prieto et al., 2016). The *vanM* gene was first identified in 2006 in a clinical isolate of VREfm from Shanghai, China (Xu et al., 2010). Although *vanM* has also been reported in Hangzhou (Sun L. et al., 2019), and in Nanjing (Zhou et al., 2020), comprehensive data on its distribution across other regions in China remain scarce. However, the dissemination mechanisms of less common gene clusters, such as *vanM*, remain poorly understood.*

These resistance genes are often located on mobile genetic elements, such as plasmids and transposons, which are associated with horizontal gene transfer (HGT). Once integrated into the bacterial genome, these genes can also be inherited vertically, contributing to their persistence in bacterial lineages. Each gene within the *van* gene cluster plays a specific role in conferring resistance to vancomycin. For example, *vanR* and *vanS* constitute a two-component regulatory system that detects vancomycin and activates the expression of resistance genes. *VanH* encodes a dehydrogenase enzyme responsible for reducing pyruvate to D-lactate. *VanA* (or *vanM*, depending on the cluster) encodes a ligase that catalyzes the formation of an altered peptidoglycan precursor, D-Ala-D-Lac, exhibiting reduced binding affinity for vancomycin. *VanX* encodes a D, D-dipeptidase that hydrolyzes the normal D-Ala-D-Ala dipeptide, thereby preventing the incorporation of vancomycin-sensitive precursors into the peptidoglycan (Stogios and Savchenko, 2020). Furthermore, *vanZA* gene, which is known to enhance resistance to teicoplanin (Arthur et al., 1995). Together, these genes orchestrate a coordinated mechanism that effectively reduces the bacterium’s susceptibility to vancomycin. The *vanA* gene cluster has been identified in diverse genetic contexts across different plasmid types and sequence types. One involves the transfer of plasmids containing identical *Tn1546* variants among strains with different clonal backgrounds, while the other is facilitated by the transposition of *Tn1546* between various plasmid types (Park et al., 2007; Novais et al., 2008). While the dissemination mechanisms of the *vanA* gene cluster have been extensively characterized, knowledge of *vanM* remains limited. The transposon type associated with *vanM* and its transfer mechanisms are poorly understood, underscoring the need for further research into its dissemination patterns across diverse strains and geographic regions.

In this study, we performed genomic characterization of VREfm isolates collected from a tertiary hospital in Jilin Province between 2019 and 2024. By integrating MLST typing, plasmid clustering (based on Mash and FastANI), and transposon structure analysis, we aimed to describe the diversity of VREfm lineages and the genetic contexts of van-carrying mobile genetic elements. Particular attention was given to the distribution of *vanA*- and *vanM*-associated plasmids and transposon variants across different sequence types and sampling periods. These results provide a genomic overview of resistance-associated mobile genetic elements in VREfm within a hospital setting.

## 2 Materials and methods

### 2.1 Bacterial strains

Clinical specimens were retrospectively collected from patients admitted to The First Hospital of Jilin University in Changchun, Jilin Province, between August 2019 to March 2024. During this period, a total of 6,223 *Enterococcus isolates* were identified in the clinical microbiology laboratory. VREfm isolates were relatively rare. Therefore, isolates included in this study were selected based on the following criteria: confirmed vancomycin resistance, sufficient DNA quality for sequencing, and availability of clinical metadata. A total of 19 VRE strains were included in the study. These isolates represent a selected subset of VREfm isolates identified during the study period, based on sequencing quality and data completeness, rather than the entirety of VREfm cases in the hospital. When multiple VRE isolates were obtained from the same patient, only the first non-duplicate isolate per infection episode was included in the genomic analysis to minimize sampling bias, avoid redundancy, and ensure that all isolates represented independent infection events. All experiments were conducted in accordance with the stringent protocols outlined in the Clinical Laboratory Standards Institute (CLSI) procedures, the official standards widely adopted by clinical laboratories worldwide (Clinical And Laboratory Standards Institute [CLSI], 2010). Bacterial strains were identified as *E. faecium* using matrix-assisted laser desorption/ionization time-of-flight mass spectrometry (MALDI-TOF MS, VITEK-MS) or the VITEK-2 automated microbial identification system. Vancomycin resistance was defined as a minimum inhibitory concentration (MIC) of vancomycin ≥ 32μg/mL, as determined by antimicrobial susceptibility testing (AST). The MICs were interpreted by the Clinical And Laboratory Standards Institute [CLSI] (2022) guidelines (Clinical And Laboratory Standards Institute [CLSI], 2022).

### 2.2 Data visualization

The Sankey diagrams and bar charts were created using Chiplot^1^ (accessed on 25 December 2024). Trends in antibiotic resistance and case distribution of *E. faecium* from 2019 to 2024 were visualized using GraphPad Prism 9.5.0. The hospitalization timeline of VREfm isolates was constructed using the ggplot2 package in R (version 4.3.3) to illustrate patient admission, sampling, and discharge dates.

### 2.3 DNA isolation, whole-genome sequencing, and assembly

For sequencing sample preparation, single bacterial colonies were picked and cultured to the logarithmic growth phase. The bacterial pellets were collected by centrifugation at 4,500 rpm for 5 min, and the culture medium was discarded. The pellets were resuspended in 1 × PBS (free of nucleases) for washing, followed by another round of centrifugation under the same conditions. After discarding the supernatant, the bacterial pellets were collected, rapidly frozen in liquid nitrogen, and shipped on dry ice to ensure sample integrity during transport. DNA extraction and whole-genome sequencing were conducted using the Illumina MiSeq and Oxford Nanopore Technology (ONT) platforms. These processes were performed by Shanghai Sangon Biological Technology Co., Ltd. The quality of the paired-end reads was assessed using FastQC 0.11.15,^2^ and low-quality reads were filtered out with Trimmomatic 0.36 (Bolger et al., 2014). For Illumina sequences, *De novo* assemblies were constructed with SPAdes 3.5.0 (Bankevich et al., 2012) and final assemblies evaluated with Quast 5.2.0 (Gurevich et al., 2013). Although SPAdes 3.5.0 was used for short-read-assisted gap closure and polishing, this step served only as a supplementary refinement. The primary genome assemblies were generated from Oxford Nanopore long-read sequencing data using Canu 1.3 (Koren et al., 2017). Additionally, the completeness and contamination of the assemblies were assessed using CheckM 1.2.2.^3^ Assembly of Nanopore single-molecule sequencing data was performed using Canu 1.3. To further investigate the circularity of the plasmids, Unicycler was used to examine whether the plasmids were circularized during assembly (Wick et al., 2017). Short-read sequencing data was incorporated, and GapFiller (Boetzer and Pirovano, 2012) was used to close gaps in the assembled contigs. *In silico* prediction using Abricate,^4^ with the Card database (Alcock et al., 2023) was conducted to search and select for isolates bearing the vancomycin resistance gene. All assemblies, including those of the 19 samples, were annotated with Prokka 1.14.6 (Seemann, 2014). Assembly quality was further evaluated based on coverage depth, N50 values, genome completeness, contamination levels (assessed using CheckM), and BUSCO scores. All assemblies met the quality criteria required for downstream comparative genomic and phylogenetic analyses.

### 2.4 Plasmid analysis

Plasmid sequences were extracted from the assembled genomes and analyzed using a combination of tools to classify and compare plasmids effectively. Pairwise distances between plasmid sequences were calculated using MASH (Ondov et al., 2016) (*k* = 21, *s* = 1,000) to group plasmids into clusters, with those having distances below 0.025 grouped for further analysis. The Mash distance cutoff (0.025) was selected based on previous studies and empirical evaluation of the present dataset to balance sensitivity and specificity in plasmid grouping (Arredondo-Alonso et al., 2021). To further validate the robustness of the clustering, FastANI (Jain et al., 2018) was applied to assess nucleotide similarity within each plasmid group. Plasmids within the same cluster showed high similarity (ANI > 95%), supporting the reliability of the clustering results. FastANI was also used to measure the overall similarity between complete plasmid sequences and to explore their evolutionary relationships. These results demonstrated the robustness and reproducibility of the plasmid clustering strategy. MOB-suite tools (Robertson and Nash, 2018), including mob-recon and mob-typer, were utilized to classify plasmids based on mobility types (e.g., conjugative, mobilizable, or non-mobilizable) and annotate replication initiation types (rep-types) for confirmation of plasmid classifications. The plasmid network was built with Cytoscape (Shannon et al., 2003). Additionally, plasmid classifications defined through these approaches were integrated with ANI-based clustering, and the heatmap.2 function in the gplots R package (version 3.0.1.1) was used to visualize the integration of plasmid types and clustering based on ANI alignment coverage. Circular comparative plots of plasmid alignments were also generated using BRIG (Blast Ring Image Generator) (Alikhan et al., 2011), which was employed to create circular comparative plots of plasmid alignments. Additionally, as a Supplementary Figure 2, Cytoscape was used to visualize the BLASTN (Altschul et al., 1990) similarity between plasmids, and Artemis Comparison Tool (ACT) (Carver et al., 2005) was employed to visualize gene comparisons between the S1P2, S7P2, and S1P3 plasmids.

### 2.5 Transposon analysis

Transposons were classified and annotated using the TnCentral database (Ross et al., 2021), with a focus on identifying transposons carrying van genes (e.g., *vanA*, *vanM*). In addition, Clinker (Gilchrist and Chooi, 2021) was used to visualize the genomic loci of transposons, allowing detailed comparative analysis of their structural organization and gene arrangements.

### 2.6 Phylogenomic analyses

Core genome alignments were performed using PhyloPhlAn to generate high-quality multiple sequence alignments (60). Maximum likelihood phylogenetic trees were constructed using IQ-TREE (Nguyen et al., 2015) (multicore version 2.1.4-beta) with optimized model parameters to achieve a final log-likelihood of -198447.976 after four optimization rounds. Ultrafast Bootstrap approximation was used to compute branch support values, with 1,000 replicates ensuring robust statistical confidence. The resulting phylogenetic trees were constructed based on core genome SNPs, with additional annotation of plasmid types and transposon variants. Visualization of the trees was performed using Itol (Letunic and Bork, 2024) (Interactive Tree of Life), incorporating metadata such as sample source, hospital department, and isolation date.

All analyses in this study were performed using commonly adopted and widely validated bioinformatics tools following standard workflows in microbial genomics. In addition, the complete bioinformatics workflow, including step-by-step instructions and scripts, is publicly available at the following GitHub repository: https://github.com/939092550lidongha-boop/VREfm-genomic-pipeline.

## 3 Results

### 3.1 Distribution and antimicrobial susceptibility patterns of enterococci in the hospital

**FIGURE 1:**
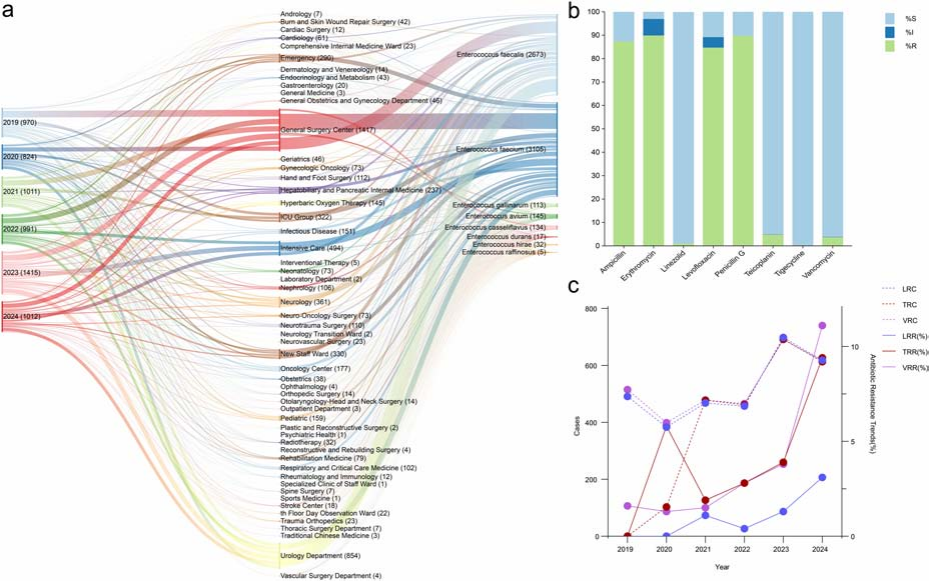
Distribution and antimicrobial resistance trends of *Enterococcus* species in a tertiary hospital (2019–2024). **(a)** Distribution of *Enterococcus* species across hospital departments. The Sankey diagram illustrates the annual distribution of *E. faecium*, *E. faecalis*, and other less common *Enterococcus* species in major clinical wards. Colors represent different years, and the width of each flow corresponds to the number of isolates. **(b)** Antimicrobial susceptibility testing results of *Enterococcus* isolates. The stacked bar chart shows the proportions of susceptible (S), intermediate (I), and resistant (R) isolates to commonly used antibiotics. **(c)** Temporal trends in antibiotic resistance and case numbers of *E. faecium* from 2019 to 2024. The left y-axis represents the number of resistant cases, and the right y-axis shows resistance rates (%). Solid lines indicate resistance rates, while dashed lines indicate the number of resistant cases. LRC, linezolid-resistant *E. faecium* case count; TRC, teicoplanin-resistant *E. faecium* case count; VRC, vancomycin-resistant *E. faecium* case count; LRR, linezolid resistance rate; TRR, teicoplanin resistance rate; VRR, vancomycin resistance rate.

During the study period (2019–2024), a total of 6,223 clinical isolates of *Enterococcus* spp. were collected from The First Hospital of Jilin University in Changchun, Jilin Province, among which *E. faecium* was the most frequently identified species, accounting for 49.88% (3,105 isolates) of the total. The distribution analysis revealed that *E. faecium* isolates were predominantly recovered from high-risk hospital departments, with higher proportions observed in departments such as the General Surgery Center (22.84%), Intensive Care Unit (ICU, 10.12%), and Urology Department (7.83%). The remaining 50.12% of isolates included *E. faecalis* and other less common species, such as *E. gallinarum* and *E. casseliflavus*, which exhibited a relatively even distribution across hospital wards (Figure 1a).

The antimicrobial susceptibility testing results revealed notable variability in antibiotic resistance rates (Figure 1b). Alarmingly high resistance rates were observed for conventional antibiotics, such as ampicillin (87.3%), erythromycin (89.88%), and penicillin G (89.73%), highlighting their limited efficacy in treating *Enterococcus* infections. Similarly, levofloxacin exhibited a resistance rate of 84.72%, with 4.45% isolates showing intermediate susceptibility. In contrast, teicoplanin and tigecycline displayed high effectiveness, with 95.14 and 100.00% susceptibility rates, respectively. Linezolid also maintained a high susceptibility rate of 98.93%, providing viable therapeutic options. Vancomycin showed a resistance rate of 3.68%, underscoring its continued utility while emphasizing the importance of vigilance in monitoring emerging resistance patterns.

A detailed analysis of antimicrobial resistance patterns showed that the vancomycin resistance rate (VRR) increased markedly from 1.6% in 2019 to 11.1% in 2024, indicating a growing clinical burden of VREfm. Correspondingly, the absolute number of vancomycin-resistant cases exhibited temporal fluctuations, rising from 515 cases in 2019 to a peak of 691 cases in 2023, followed by a decline to 603 cases in 2024. Similar temporal trends were observed for teicoplanin- and linezolid-resistant cases, both of which reached their highest levels in 2023 (692 and 698 cases, respectively) and decreased in 2024. Despite year-to-year variability, the overall resistance rates demonstrated an increasing trend during the study period. In contrast, resistance rates to conventional antibiotics, including penicillin G, ampicillin, and erythromycin, remained consistently high with only minor variations. These findings reflect the increasing clinical burden of multidrug-resistant *Enterococcus*, particularly VRE, in this hospital setting (Figure 1c).

### 3.2 Clinical and antimicrobial characteristics of VRE patients

In this study, a total of 19 VREfm isolates were collected from patients at the First Hospital of Jilin University between 2019 and 2024. These isolates were obtained from different clinical departments and various specimen types, with urine being the most common source. Detailed clinical and epidemiological information of the isolates is summarized in Table 1.

**TABLE 1:** Clinical characteristics and sources of the 19 VREfm isolates.

Sample	Isolation date	Sample source	Department	Bed ID
S1	20190823	Blood	General Surgery Center—Hepatobiliary and Pancreatic Surgery Department (HPB)	1256-1
S2	20240201	Urine	Hyperbaric Oxygen Therapy (HBOT)	816
S3	20210317	Urine	Urology Department (URO)	2147-3
S4	20200922	Urine	Neurotrauma Surgery (NTS)	2431-1
S5	20231225	Urine	Respiratory and Critical Care Medicine (RCCM)	RICU08
S6	20231217	Urine	Hyperbaric Oxygen Therapy (HBOT)	816
S7	20220527	Urine	Intensive Care Medicine (ICM)	ICU206
S8	20240305	Urine	Hyperbaric Oxygen Therapy (HBOT)	830
S9	20240221	Urine	Hyperbaric Oxygen Therapy (HBOT)	809
S10	20211221	Secretion	ICU Group (ICU)	ICU115
S11	20231217	Drainage fluid	General Surgery Center—Hepatobiliary and Pancreatic Surgery Department (HPB)	1297-2
S12	20240131	Cerebrospinal fluid	Neuro-Oncology Surgery (NOS)	1946-1
S13	20200714	Urine	Emergency Internal Medicine (EIM)	EICU16
S14	20240121	Purulent fluid	Infectious Disease (ID)	182-03
S15	20240129	Urine	Intensive Care Medicine (ICM)	ICU113
S16	20240128	Urine	Hyperbaric Oxygen Therapy (HBOT)	815
S17	20210325	Urine	Neurology (NEU)	202-03
S18	20240124	Catheter blood	Pediatric ICU (PICU)	NCU-09
S19	20201030	Urine	ICU Group (ICU)	ICU315

The antimicrobial susceptibility profiles of the 19 VREfm isolates are presented in Supplementary Table 1. All isolates demonstrated resistance to vancomycin, with minimum inhibitory concentration (MIC) values ≥ 32μg/mL, meeting the CLSI-defined criteria for vancomycin resistance (Clinical And Laboratory Standards Institute [CLSI], 2022). Antimicrobial susceptibility testing revealed that all isolates were highly resistant to penicillin (MIC ≥ 64 μg/mL), ampicillin (MIC ≥ 32 μg/mL), erythromycin (MIC ≥ 8 μg/mL), and levofloxacin (MIC ≥ 8 μg/mL). Resistance to teicoplanin was variable, with MIC values ranging from 32 μg/mL to ≥ 64 μg/mL, while tigecycline remained highly effective, with most isolates exhibiting MIC values ≤ 0.12μg/mL. Resistance to linezolid appeared in a subset of isolates, with MIC values ranging from 2 μg/mL to ≥ 8 μg/mL. These findings highlight these isolates’ multidrug-resistant (MDR) nature, posing significant challenges for clinical management.

Whole genome sequencing and analysis using the CARD database confirmed the presence of *van* genes in all 19 isolates (Supplementary Table 2). Among these, three isolates carried partial *van* gene clusters, including *vanHM*, *vanYM*, and *vanXM*. nine isolates harbored complete *vanA*-associated resistance gene clusters, comprising *vanHA*, *vanA*, *vanXA*, *vanYA*, *vanRA*, *vanSA*, and *vanZA*. Additionally, seven isolates carried *vanA* and *vanM*-associated resistance gene clusters, including *vanHA*,*vanA*,*vanXA*, *vanYA*, *vanRA*, *vanSA*, *vanZA*, and the *vanM* components *vanYM*, *vanSM*, and *vanRM*. These findings demonstrate the widespread prevalence of van resistance genes, particularly those associated with *vanA* and *vanM* clusters, among the studied isolates, underscoring the urgent need for targeted infection control strategies to mitigate the dissemination of multidrug-resistant and highly transferable resistance determinants.

### 3.3 Temporal distribution and hospitalization timeline

**FIGURE 2:**
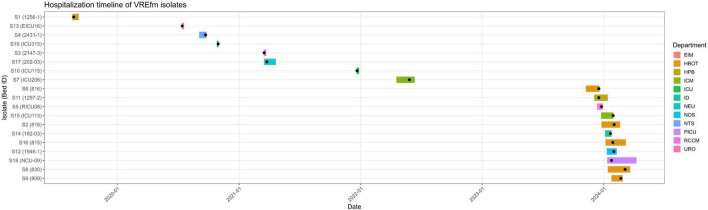
Hospitalization timeline of patients with VREfm isolates. Each horizontal bar represents the hospitalization period of an individual patient from admission to discharge. The colored bars indicate the clinical department where the isolate was obtained. Black dots represent the sampling date of the VREfm isolate.

To provide a descriptive overview of VREfm cases, the hospitalization timelines of patients carrying VREfm isolates were reconstructed (Figure 2). Each bar represents the hospitalization period from admission to discharge, while black dots indicate the sampling time of the isolate.

The timeline shows sporadic cases between 2019 and 2022, followed by a higher number of sequenced isolates in 2023–2024. However, this pattern should be interpreted with caution, as sequencing and sampling were not evenly distributed across the study period.

### 3.4 Plasmidome characterization and network-based classification using third-generation sequencing

**FIGURE 3:**
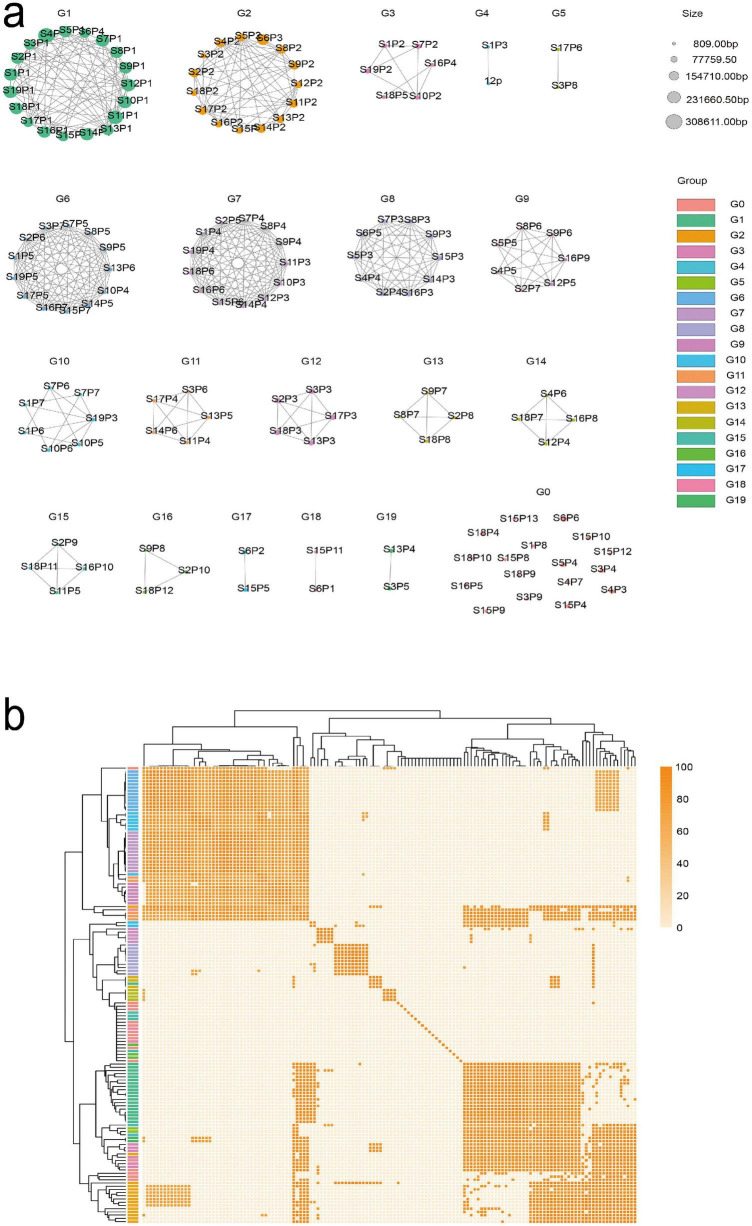
Clustering and genetic similarity analysis of plasmid types. **(a)** Plasmids are grouped into distinct clusters based on sequence similarity, as determined by Mash pairwise comparisons (*k* = 21, s = 1,000). The clustering illustrates clear modularity among plasmid types. **(b)** A heatmap generated from FastANI results, displaying ANI and coverage for pairwise comparisons between plasmid sequences. This visualization provides a detailed representation of genetic similarity and alignment metrics, enabling precise differentiation among plasmid types.

This study employed third-generation sequencing technology to extract plasmid sequences from each sample, as Illumina sequencing could not fully assemble plasmids. Each plasmid was named according to its isolate and plasmid number (e.g., S1P2 represents plasmid 2 from isolate S1). The plasmids were subsequently grouped and classified using MOB-suite, Mash, and FastANI (Figure 3). The quality of the genome assemblies was evaluated using long-read sequencing depth and multiple quality metrics. Detailed information, including genome size, GC content, sequencing depth, completeness, contamination, N50, and BUSCO results, is summarized in Supplementary Table 3, supporting the reliability of genome and plasmid reconstruction. Sample 12 (12p) was analyzed based on second-generation sequencing, as third-generation sequencing data revealed the loss of its resistance genes. MOB-suite served as a powerful tool for the initial clustering and typing of plasmids based on features such as replicon type, mobility, and other genetic characteristics. Supplementary Table 4 shows the features of the 142 plasmids found among the 19 fully sequenced genomes. Subsequently, complementary analyses with Mash (based on k-mer analysis) and FastANI (calculating average nucleotide identity, ANI) enabled rapid comparisons of whole-genome similarities.

Using Mash (*k* = 21, *s* = 1,000), pairwise comparisons were performed on plasmid sequences (*n* = 142), encompassing plasmids extracted from a combination of third generation and second-generation sequencing. The resulting similarity data were integrated into a network, with a cutoff value of 0.025 defining edges, leading to the identification of 19 independent groups within the network (Group_1-Group_19), while Group_0 was identified as a singleton, indicating that it did not share sufficient similarity with any other group. Resistance genes were predominantly concentrated in Groups_2, Groups_3, Groups_4, and Groups_5. Supplementary Table 5 provides detailed Mash comparisons.

To gain deeper insights into the modularity and similarity of these plasmid types, we determined ANI values using FastANI. This analysis evaluated ANI coverage and identity values for aligned regions in pairwise comparisons of complete plasmid sequences. Plasmids classified within the same plasmid type demonstrated an average alignment coverage exceeding 90%, indicating high sequence similarity. Supplementary Table 6 provides detailed FastANI results.

BLAST analysis was conducted on plasmid sequences containing resistance genes from Groups_2, Groups_3, Groups_4, and Groups_5 against publicly available plasmid databases to explore the relationships among the identified plasmids further. The analysis revealed that Group_2 plasmids shared high sequence similarity with reference plasmids LC495616.1 and CP019208.1. Group_3 plasmids exhibited high similarity to CP130846.1 and CP131096.1, indicating that these plasmids are conserved and may represent conserved plasmid backbones associated with resistance genes. In contrast, intra-group comparisons were performed for plasmids in Groups_4 and Groups_5. In Group_4 (S1P3 and 12p), showed high intra-group similarity. Still, they had low coverage compared to external reference sequences, suggesting that these plasmids might represent fragmented or incomplete sequences. By performing Artemis comparisons of S1P2, S7P2, and S1P3, we hypothesize that S1P3 may represent a fragment derived from a larger plasmid (Supplementary Figure 1). Based on their shared structural elements, S1P3 and S1P2 are hypothesized to be a fragment derived from S7P2. To provide a comprehensive background for analyzing the structural and sequence similarities, S7P2, a complete reference plasmid, was used for comparison. To further understand the structural variations of plasmids, we performed BLASTN alignments, retaining only those results with an identity of ≥ 90% and a query coverage (qcov) of ≥ 90%. The similarity between plasmids was visualized through a network diagram generated in Cytoscape (Supplementary Figure 2). We observed that some plasmids exhibited high genomic structural similarity, while others displayed significant differences. Notably, plasmids without replicons (indicated by black font) were identified, suggesting that these plasmids may have undergone fragmentation, resulting in the loss of replicons. Plasmids lacking replicons may not replicate autonomously and may not replicate autonomously and may therefore represent incomplete or non-self-transmissible elements. This phenomenon might be closely linked to the adaptive cost of plasmids (Rodríguez-Beltrán et al., 2022), where bacteria may discard unnecessary genetic material through plasmid recombination or fragmentation, thereby reducing physiological burdens.

**FIGURE 4:**
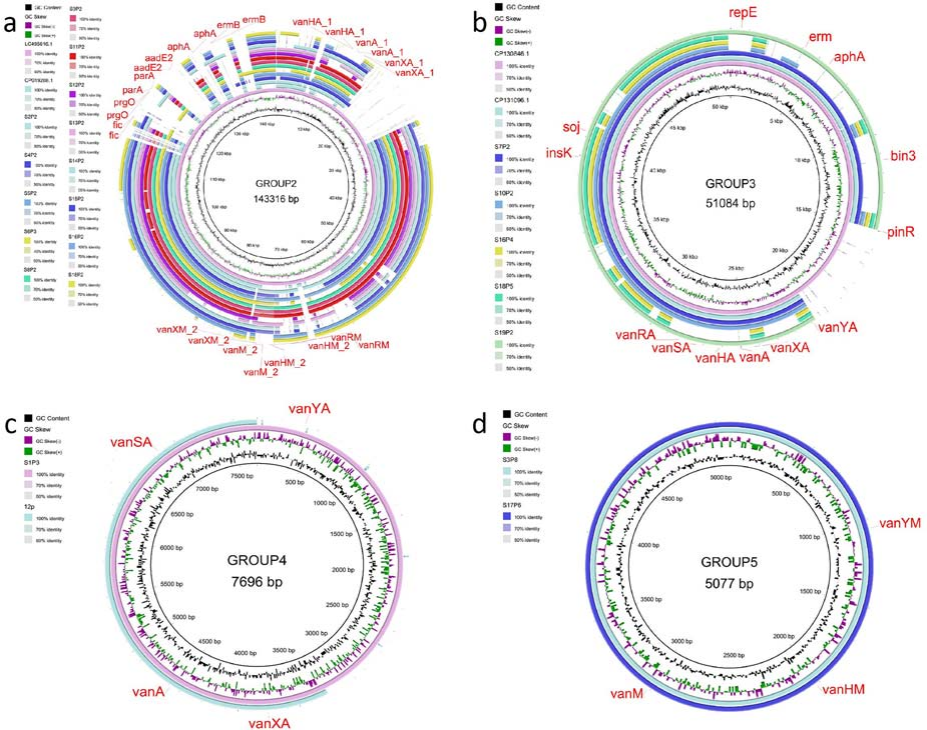
Comparative analysis of plasmid groups using circular maps. **(a)** Group 2 Plasmids: Show high sequence similarity with reference plasmids LC495616.1 and CP019208.1, highlighting their potential role in the dissemination of resistance genes. **(b)** Group 3 Plasmids: Exhibit high similarity to reference plasmids CP130846.1 and CP131096.1, indicating that these plasmids are highly conserved and may play a key role in the spread of resistance genes. **(c)** Group 4 Plasmids: Two plasmids (∼7,956 bp) demonstrate high intra-group similarity but low coverage compared to external reference sequences, suggesting the presence of fragmented or incomplete sequences. **(d)** Group 5 Plasmids: Two plasmids (∼5,077 bp) associated with *vanM* resistance genes show high intra-group similarity but low coverage against external reference sequences, which may reflect transposon-mediated events or plasmid fragmentation.

Similarly, the two plasmids in Groups_5 (S3P8 and S17P6), associated with *vanM* resistance genes, also displayed high intra-group similarity but low coverage against external reference sequences. This low coverage may reflect structural variation or possible plasmid fragmentation. Using BRIG (Figure 4), circular comparative maps were generated to visually depict conserved and variable regions within and between these plasmid groups.

### 3.5 Classification of Tn1546 transposons in VRE isolates

**FIGURE 5:**
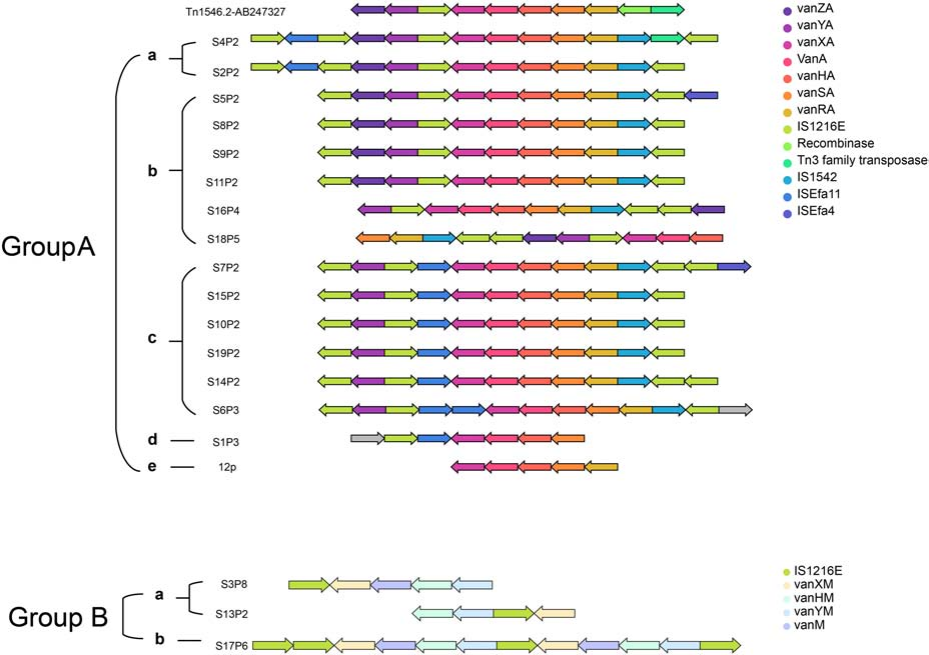
Classification and genetic structures of *Tn1546* transposons in VRE isolates. The transposons are categorized into Group A and Group B, where Group A is further divided into five subgroups (a, b, c, d, e), while Group B consists of only two subgroups (a, b). Each row represents a distinct *Tn1546* variant, with arrows indicating the relative positions and orientations of genes. Color coding is used to differentiate functional gene categories. Group A exhibits a relatively conserved gene arrangement, with minor deletions or mutations in certain subgroups (such as b and c), whereas Group B shows significant gene deletions or rearrangements near *IS1216E*, resulting in a more diverse genetic structure.

Next, the study focused on an in-depth analysis of *Tn1546* transposons, which play a key role in VRE. As shown in Figure 5, the analysis aimed to uncover these transposons’ genetic structure, variability, and evolutionary patterns.

The *Tn1546* transposons are divided into Group A and Group B, further categorized into subgroups (Aa, Ab, Ac, Ad, Ae, Ba, and Bb) based on specific genetic arrangements. Core resistance genes, including *vanA*, *vanXA*, *vanHA*, and others, are represented as colored arrows in the figure, showcasing their essential role in vancomycin resistance. Additionally, transposable elements such as *IS1216E*, *IS1542*, *ISEfa11*, and *ISEfa4* are depicted, highlighting their association with structural variation of these resistance gene clusters.

Group A consists of multiple subgroups (a, b, c, d, and e) with high genetic similarity. Subgroup Aa, for example, corresponds to the reference sequence *Tn1546.2-AB247327*, illustrating the canonical genetic structure of these transposons. In contrast, subgroups Ab to Ae exhibit structural rearrangements and include additional elements, such as *ISEfa11* or *vanZA*, are associated with increased genetic variability. Group B, on the other hand, displays distinct genetic structures with fewer associated elements, indicating significant evolutionary divergence from Group A. The presence of unique resistance determinants within Group B, such as *vanXM* and *vanYM*, further highlights its distinct genetic features.

### 3.6 Phylogenomic relationships and temporal genomic variation

**FIGURE 6:**
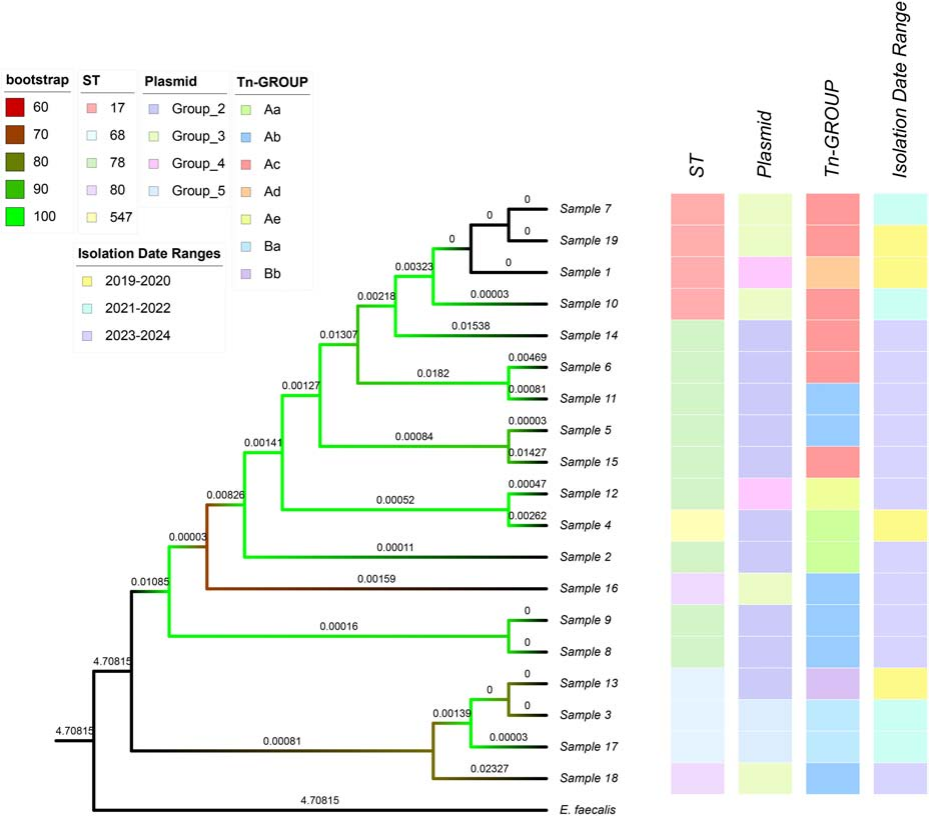
The core genome-based phylogenetic tree represents 19 VRE isolates collected from 2019 to 2024. The tree integrates metadata on sequence types (ST), plasmid groups (Group_2, Group_3, Group_4, Group_5), and T*n1546* transposon variants (Aa, Ab, Ac, Ad, Ae, Ba, Bb). Sequence types and isolation years are visually differentiated using color coding, while branch annotations indicate the plasmid group and transposon variant associated with each isolate.

This comprehensive analysis of 19 VRE isolates using PubMLST for MLST typing identified multiple sequence types (STs), including ST17, ST68, ST78, ST80, and ST547 (Figure 6). We constructed a core genome-based phylogenomic tree and integrated metadata on plasmid types and *Tn1546* variants to describe the genetic relationships among isolates and their associated plasmid types and transposon variants.

ST17 primarily appeared during 2019–2022 and was linked to plasmids from Group_3 (Sample 7, Sample 19 and Sample 10) and Group_4 (Sample 1); Transposon types included Ac (Sample 7, Sample 19 and Sample 10) and Ad (Sample 1). ST68 was also concentrated during 2019–2022. It was associated with plasmids from Group_5 (Sample 3 and Sample 17) and Group_2 (Sample 13), with transposon types of Ba (Sample 3 and Sample 17) and Bb (Sample 13); ST78 became the dominant sequence type during 2023–2024. It was primarily linked to plasmids from Group_2 (Sample 4, Sample 6, Sample 11, Sample 5, Sample 15, Sample 2, Sample 8, and Sample 9) and secondarily to Group_4 (Sample 12). This sequence type exhibited diverse transposon types, including Aa (Sample 12), Ab (Sample 5, Sample 8, Sample 9 and Sample 11), Ac (Sample 15 and Sample 6), and Ae (Sample 12); ST80 (Sample 16 and Sample 18) emerged during 2023–2024, associated with Group_3 plasmids and transposon type Ab. ST547 (Sample 4) was identified in one sample during 2019–2020, linked to a Group_2 plasmid with transposon type Aa. These findings illustrate the diversity of VREfm lineages and the association between sequence types, plasmid groups, and transposon variants in this hospital collection. To statistically evaluate temporal changes in population structure, the distribution of STs was compared between the early (2019–2022) and late (2023–2024) periods using Fisher’s exact test for a 2 × 5 contingency table. A difference in ST composition was observed between the two periods (*P* < 0.001; Supplementary Table 7). Based on the integration of MLST typing, plasmid grouping, and Tn1546 variant analysis, most isolates shared consistent combinations of sequence types, plasmid groups, and transposon variants, while a small number of isolates exhibited different genomic configurations. These observations reflect heterogeneity in the genetic contexts of van-carrying mobile elements among the isolates (Supplementary Table 8). Differences in sequence type composition and associated plasmid and transposon characteristics were observed between isolates collected in 2019–2022 and those from 2023 to 2024 (Figure 6).

## 4 Discussion

The emergence and occurrence of VRE have become significant public health concerns, particularly in parts of Asia, Europe, and North America (Deshpande et al., 2007; Chang et al., 2010; Boumasmoud et al., 2022). In China, particularly in the eastern provinces, the prevalence of VRE has increased in recent years, which may be associated with the expansion of livestock farming and the overuse of antibiotics in the region. The northeastern provinces, as major agricultural hubs of the country, have seen extensive use of antibiotics in both livestock production and crop cultivation, which may have contribute to the emergence and occurrence of antibiotic-resistant pathogens (Manyi-Loh et al., 2018). Furthermore, the relatively lower levels of healthcare infrastructure, including medical equipment, healthcare services, sanitation, and disease prevention measures, compared to more developed regions, such as Beijing, Shanghai, and Shenzhen, may contribute to the increased occurrence of VRE in this area. Particularly during the COVID-19 pandemic, the overuse and misuse of antibiotics have further exacerbated the spread of drug-resistant pathogens (Pandak et al., 2023; Sokolović et al., 2023), suggesting that the increase in VRE may be associated with to this backdrop. Given these concerning trends, a deeper understanding of the genomic epidemiology and genetic diversity of VRE in hospital settings is crucial.

This study represents the first genomic investigation of VRE genomic characterization and antimicrobial resistance characteristics in a tertiary hospital in Jilin Province, providing valuable insights into the mechanisms of resistance gene dissemination and plasmid evolution. Our study is consistent with previous research, which indicates that VRE transmission occurs through both clonal expansion for vertical transmission and HGT mediated by plasmids and transposons (Arredondo-Alonso et al., 2021). To investigate these genomic characteristics, we conducted a genomic study focusing on MLST typing, plasmid diversity, and transposon variability in VRE isolates.

Since genomic approaches have demonstrated clear advantages over traditional molecular typing methods in the epidemiological surveillance of nosocomial pathogens (Van Goethem et al., 2019), they have become indispensable tools for studying genetic diversity, transmission dynamics, and resistance mechanisms of hospital-associated infectious agents. Epidemiological studies on VRE in Chinese hospitals have gradually increased in recent years. According to a study by Pan et al. (2024), *E. faecium* exhibits generally higher drug resistance than *E. faecalis*, with the primary resistance mechanisms of VRE being mediated by the *vanA* and *vanM* genes. Both *E. faecium* and *E. faecalis* were predominantly found in urine samples from hospitals, with *E. faecium* demonstrating more significant multi-drug resistance. This is consistent with our findings, where *E. faecium* was also the predominant species observed in hospital urine samples (13/19), exhibiting high levels of multi-drug resistance. In this study, differences in the distribution of VRE were observed across departments and periods, while variations in resistance rates to vancomycin were noted across the study period, accompanied by a marked rise in resistance to commonly used antibiotics such as penicillin, ampicillin, and erythromycin. A study conducted at Peking Union Medical College Hospital analyzed the drug resistance and molecular characteristics of VRE and reported that all VREfm isolates carried the *vanA* gene, and 11.9% carried the *vanM* gene (Sun H. L. et al., 2019). Furthermore, all VREfm isolates resisted ampicillin but remained sensitive to daptomycin, linezolid, and teicoplanin. These findings align closely with the results of this study, where all 19 VREfm isolates demonstrated resistance to vancomycin, with MIC values ≥ 32μg/mL, as well as high resistance to penicillin, ampicillin, erythromycin, and levofloxacin. These findings suggest that specific STs are linked to distinct plasmid and transposon types, which are associated with in VRE genetic diversity.

To further investigate resistance genetic characteristics, we examined the sequence variations in plasmids and transposons. In our study, we found that some plasmids containing resistance genes lack replicators, while some plasmids with replicators do not carry resistance genes. Plasmid clustering analysis identified 19 distinct groups (Group_1 to Group_19), with Group_2 and Group_3 showing strong associations with vancomycin resistance. Some resistance plasmids within these groups exhibited replicon loss, potentially due to recombination or fragmentation events. This phenomenon may be related to gene recombination and replicon loss during plasmid recombination or structural variation or shedding. Plasmid fragmentation may serve as an possible explanation for the dynamic regulation of resistance genes (Beaudier et al., 2024). Heaton et al. reported the transfer of the *Tn1546* element from a non-conjugative plasmid to a conjugative plasmid, mediated by the flanking *IS1216* elements (Heaton et al., 1996). A similar phenomenon was observed regarding the transposition of *Tn1546* between different plasmids, specifically about the *vanA* and *vanM* gene clusters. Our samples observed that identical transposon types, including those carrying the *vanA* and *vanM* gene clusters, could exist in different plasmid types while still being observed within the same clonal background. This suggests that the *vanA* and *vanM* gene clusters within transposons may exist in multiple forms within the same bacterial population and are associated with the distribution of resistance genes through different plasmid contexts between plasmids.

The relationships between different sequence types, plasmids, and transposons, revealing their associations in resistance-related genetic contexts resistance in VRE. The distribution of the *vanA* gene cluster can occur at a plasmid level in which both the plasmid type and *Tn1546* variant are horizontally disseminated, as observed in South Holland between 2014 and 2015 (Di Sante et al., 2017). Vertically, it is passed down within clonal isolates that have identical plasmid types and variants of *Tn1546*. HGT shapes bacterial evolution by enabling bacteria to adapt to new ecological niches and thrive in diverse environments (Wiedenbeck and Cohan, 2011). Plasmids are one of the primary vectors for HGT, carrying essential traits such as antibiotic resistance, virulence, and metabolic genes (Rodríguez-Beltrán et al., 2021). However, plasmids impose a physiological burden on bacteria, and in the absence of selective advantages conferred by plasmid-encoded traits, their presence can reduce bacterial fitness (San Millan and MacLean, 2017). The adaptive cost of plasmids is a major limitation to their transmission and persistence within bacterial populations (Harrison and Brockhurst, 2012). Notably, in Sample 12 (12p), plasmid-mediated resistance gene loss was observed in third generation sequencing data, highlighting the dynamic nature of plasmid stability and its impact on resistance genetic variation. Transposons are another crucial component of HGT, playing an important role in gene transfer between plasmids and chromosomes (Tokuda and Shintani, 2024). These mobile genetic elements facilitate the rapid spread of antibiotic resistance genes, helping bacteria adapt to environmental pressures.

In this study, genomic relationships among isolates were explored by integrating phylogenetic clustering, plasmid similarity, and transposon structural analysis. Isolates sharing the same sequence type and exhibiting minimal core-genome SNP differences showed high genomic similarity. In addition, similar resistance plasmid backbones or conserved *Tn1546* variants were observed across some genetically distinct isolates. These observations highlight the diversity of genomic configurations involving sequence types, plasmids, and transposons within the dataset. Differences in the distribution of sequence types, plasmid groups, and transposon variants were observed between isolates collected in different periods.

ST17 predominantly appeared between 2019 and 2022 and was associated with GROUP_3 and GROUP_4 plasmids, exhibiting partial transposon loss. ST68, which carried the *vanM* gene cluster in three samples, was also concentrated during this period and associated with GROUP_2 and GROUP_5 plasmids, with relatively stable transposon types. The presence of *vanM* suggests its potential role in being associated with multidrug resistance within specific plasmid types. In our dataset, *vanM*-associated isolates were primarily detected in the earlier period (2019–2022), whereas *vanA* predominated among the sequenced isolates collected in 2023–2024. This temporal pattern is noteworthy and may suggest a shift in the dominant van-associated mobile genetic elements. Although the *vanM* gene cluster has been previously reported in several regions of China, the novelty of the present study lies in the genomic characterization of *vanM*-associated mobile genetic elements, including their plasmid contexts, transposon structures, and distribution across multiple sequence types within a longitudinal hospital cohort. These analyses provide further evidence into the potential genetic diversity patterns and evolutionary patterns of *vanM* in clinical settings. ST78 was more frequently observed among the sequenced isolates collected during 2023–2024, demonstrating extensive transposon diversity, which may indicate strong adaptability and an enhanced ability to carry resistance genes. These findings highlight the importance of integrating genomic surveillance into routine infection control strategies. In Nanjing Drum Tower Hospital (Zhou et al., 2020) and Peking Union Medical College Hospita (Sun L. et al., 2019), which identified ST78 as the leading sequence type in VREfm, with a decreasing prevalence trend over the years. Additionally, *vanA* and *vanM* were identified as the main determinants of vancomycin resistance in their study, supporting the presence of *vanM* in our dataset on resistance gene distribution.

*VanA-type* resistance was also investigated in Australia using a combination of short- and long-read sequencing (Lee R. S. et al., 2018). The study showed the presence of several *vanA* plasmid types, dominant in each ST group, with distinct *Tn1546* variants. This revealed that in Australia, the emergence of *vanA*-type resistance most likely occurred through multiple introductions of different clones, suggesting that multiple mechanisms may contribute to the observed genomic patterns of the *vanA* gene cluste. Our study also observed complex dissemination patterns *vanA* and *vanM* gene clusters across multiple sequence types, further confirming the intricate mechanisms of resistance gene transfer.

ST80, although limited to two samples, was associated with GROUP_3 plasmids and a singular transposon type, showing similarities to findings by Pinholt et al. (2019), where ST80 was linked to early local outbreaks and the clonal spread of the *vanA* gene cluster. Despite the small number of samples, the presence of ST80 in our dataset suggests that it may act as an emerging sequence type under specific selective pressures.

ST547 was more isolated in its temporal distribution without significant distinguishing features. Overall, the temporal distribution of different STs and the diversity and dynamics of plasmids and transposons play a crucial role in the distribution of resistance genes and bacterial environmental adaptability. Early isolates were primarily confined to ST17 and ST68 lineages, whereas ST78 became dominant in the later study period. This temporal pattern, together with increasing plasmid and *Tn1546* diversity, indicates a differences from predominantly genomic patterns associated with specific STs to dissemination increasingly influenced by variation in mobile genetic elements.

The hospitalization timeline analysis further indicated that VREfm cases were relatively sporadic during the early study period (2019–2022), whereas a higher number of sequenced isolates was observed in recent years (2023–2024). This temporal pattern may imply a change in the genomic patterns of VREfm within the hospital and is broadly consistent with the genomic observations suggesting a transition from early distribution of sequence types dominated by ST17 and ST68 to the later emergence and predominance of ST78 associated with diverse plasmid backbones and *Tn1546* variants.

From an infection control perspective, our findings have several important implications. First, the observed differences from early genomic patterns to later variation associated by plasmids and transposons highlights the need for enhanced genomic surveillance focusing not only on strain typing but also on mobile genetic elements. Routine monitoring of resistance plasmids and transposon variants may enable earlier detection of emerging resistance genetic variation pathways.

Second, the temporal clustering of specific sequence types, particularly the emergence and dominance of ST78 in recent years, suggests that targeted surveillance and infection prevention measures in high-risk wards, such as intensive care units and departments with high antibiotic exposure, may be especially important. Strengthening screening strategies, contact precautions, and environmental disinfection in these settings could help limit occurrence.

Third, given the high resistance rates to commonly used antibiotics and the preserved susceptibility to linezolid, teicoplanin, and daptomycin in our study, antimicrobial stewardship programs should emphasize rational use of these last-line agents to prevent the emergence of further resistance. In particular, minimizing unnecessary use and optimizing treatment duration may reduce selective pressure and limit the occurrence of resistant strains.

Finally, the dynamic nature of plasmid fragmentation and transposon-mediated resistance gene transfer observed in this study underscores the importance of continuous genomic monitoring to support real-time infection control and guide hospital policies.

This study offers valuable insights into the occurrence patterns, genomic diversity, and resistance mechanisms of VREfm. These results suggest that variation of resistance determinants mediated by mobile genetic elements may be associated with the observed genomic diversity. However, this study has several limitations that should be considered when interpreting the findings. First, only a subset of VREfm isolates recovered during the study period was subjected to whole-genome sequencing. In addition, the number of sequenced isolates was unevenly distributed across different years, which may have influenced the observed temporal patterns in sequence types, plasmid groups, and van gene distribution. Therefore, the observed genomic patterns provide insights into the temporal evolution and occurrence of VREfm in this hospital setting, although further validation in larger cohorts is warranted. The statistically significant differences in sequence type distribution between study periods, together with the concordant genomic evidence of plasmid and transposon diversity, suggest that the observed temporal differences should be interpreted with caution, as they may be influenced by sampling variability.

Second, although genomic similarities in sequence types, plasmid structures, and transposon elements were identified, direct functional validation of gene variation across isolates was not performed. Thus, the conclusions regarding potential variation are based on genomic inference rather than experimental confirmation. Nevertheless, the integration of short- and long-read sequencing enabled high-resolution characterization of resistance determinants and mobile genetic elements, providing insights into the genetic variation of resistance determinants that are difficult to achieve using conventional surveillance approaches. Despite this limitation, the combined genomic and temporal analyses provide supportive evidence for the observed genomic characteristics.

Future studies integrating genomic, clinical, and epidemiological data in larger prospective and multicenter cohorts are needed to further validate these findings and better understand the genomic characteristics and evolutionary mechanisms of VREfm.

## 5 Conclusion

In conclusion, this study provides exploratory genomic insights into the diversity and relatedness of resistance determinants in VREfm within a single hospital setting. The findings highlight the coexistence of different sequence types, plasmid groups, and transposon variants, reflecting the complexity of the genomic landscape of VREfm. However, larger multicenter studies integrating genomic, epidemiological, and functional data are required to further validate these observations.

## Data Availability

The datasets presented in this study can be found in online repositories. The names of the repository/repositories and accession number(s) can be found at: https://www.ncbi.nlm.nih.gov/genbank/. The genome assemblies are publicly available in GenBank under BioProject PRJNA1227402. The corresponding BioSample accession numbers are SAMN46982249 to SAMN46982286.

## Footnotes

1
https://www.chiplot.online/

2
https://www.bioinformatics.babraham.ac.uk/projects/fastqc/

3
https://ecogenomics.github.io/CheckM/

4
https://github.com/tseemann/abricate
