# Supplementary figures and images for: Genomic characterization of vancomycin-resistant Enterococcus faecium and van-carrying mobile genetic elements in a tertiary hospital in northeastern China
Source: Front Microbiol. 2026 Apr 10;17:1804495. doi: 10.3389/fmicb.2026.1804495 (PMC13106145; doi:10.3389/fmicb.2026.1804495)

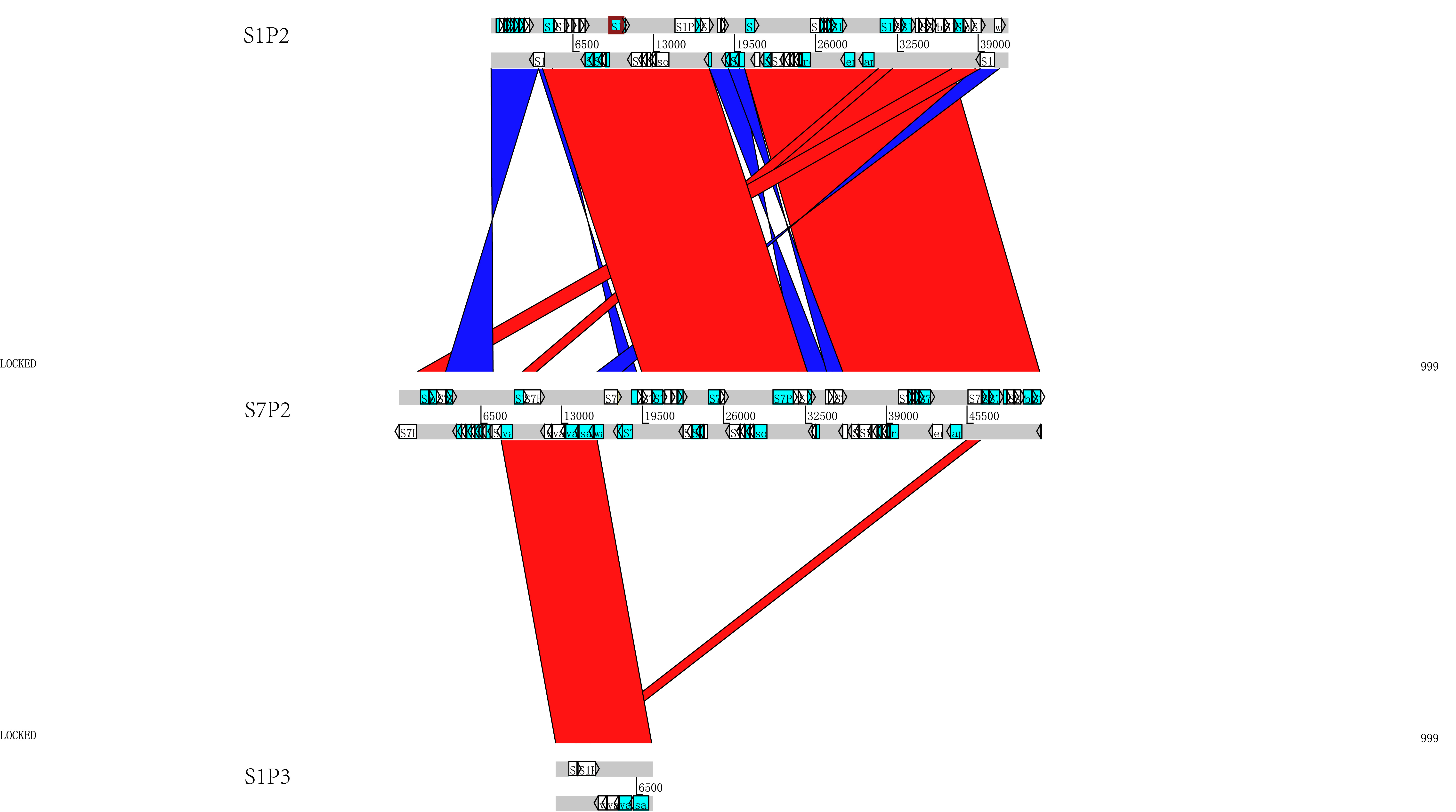

Supplement: Supplementary file 9 [file Image_1.jpeg]

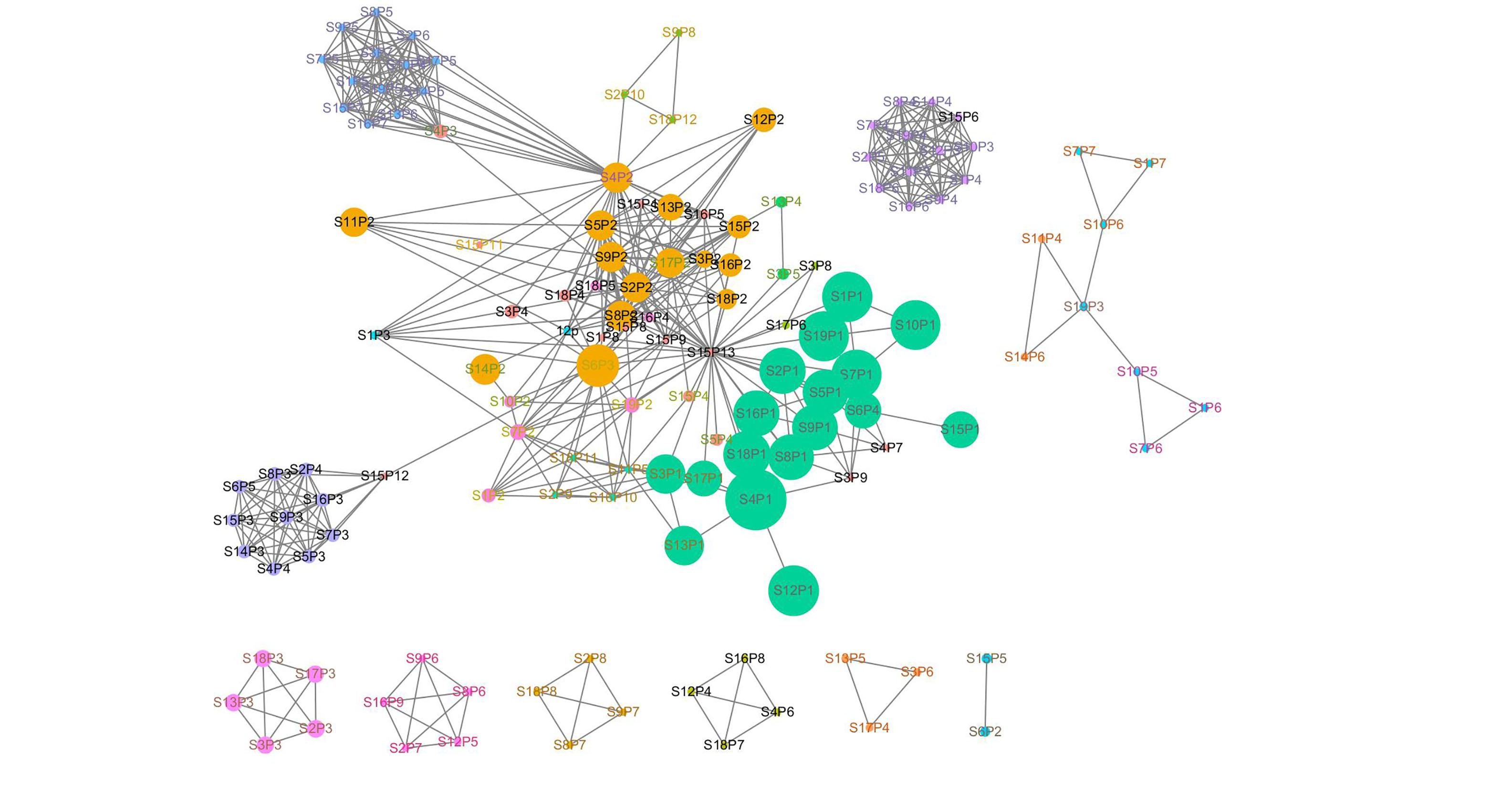

Supplement: Supplementary file 10 [file Image_2.jpeg]
